# In Vitro Human Fetal Pancreatic Islets to Redefine Pancreatic Research

**DOI:** 10.7759/cureus.43244

**Published:** 2023-08-09

**Authors:** Sipra Rout, Soosai Manickam Amirtham, Mythraeyee Prasad, Anne George Cherian, Sandya Rani B, Yesudas Sudhakar, Neetu Prince

**Affiliations:** 1 Anatomy, All India Institute of Medical Sciences, Bhubaneswar, IND; 2 Physiology, Christian Medical College and Hospital, Vellore, IND; 3 Anatomy, Velammal Medical College Hospital and Research Institute, Madurai, IND; 4 Community Medicine, Christian Medical College and Hospital, Vellore, IND; 5 Research, Christian Medical College and Hospital, Vellore, IND; 6 Biochemistry, Christian Medical College and Hospital, Vellore, IND

**Keywords:** confocal, cell clustering, insulin release, islet regeneration, fetal pancreas

## Abstract

Background:In vitro studies with human fetal islets of different gestational ages (GA) would be a great tool to generate information on the developmental process of the islets as this would help to recontextualize diabetes research and clinical practice. Pancreatic islets from human cadavers and other animal species are extensively researched to explore their suitability for islet transplantation procedure, one of the upcoming treatment strategies for insulin-dependent diabetes mellitus. Although human fetal islets are also considered for islet transplantation, ethical issues and limited knowledge constraints their use. The fetal islets could be explored to address the information lacunae on the maturity process of pancreatic islets and the endocrine-exocrine signaling mechanisms.

Aim: This study aimed to assess the feasibility of isolating viable islets and study the cytoarchitecture of the fetal pancreas of GA 22-29 weeks, not reported otherwise.

Methodology: Pancreas obtained from the aborted fetuses of GA 22-29 weeks were subjected to collagenase digestion and were further cultured to determine the viability in vitro. Parameters assessed were expression of markers for endocrine cell lineages and insulin release to glucose challenge.

Results: Islets were viable in vitro and islets were shown to maintain cues for post-digestion re-aggregation and expansion in culture. The immunofluorescent staining showed islets of varying sizes, homogenous cell clusters aggregating to form heterogenous cell clusters, otherwise not reported for this GA. On stimulation with different concentrations of glucose (2.8 and 28 mM), the fetal islets in the culture exhibited insulin release, and this response confirmed their viability in vitro.

Conclusion: Our findings showed that viable islets could be isolated and cultured in vitro for further in-depth studies to explore their proliferative potential as well as for the identification of pancreatic progenitors, a good strategy to take forward.

## Introduction

The possibility of using pancreatic islets for transplantation in insulin-dependent diabetic mellitus recipients had intrigued researchers for a substantial period now. The prospects of using fetal pancreatic tissue as a suitable and feasible alternative gained pace in the field when the paucity of organ donors started to become an impeding factor in islet transplantation [1-3]. The high immunogenicity and poor self-renewal of the adult pancreas might potentially be addressed using human fetal pancreatic precursor tissues [1]. Pre-clinical models provide evidence for using fetal tissues due to proliferative potential [4-6]. In 2004, Djordjevic’s team could temporarily restore the glucose levels with human fetal islet transplantation in type 1 diabetic patients [7]. However, considering the ethical issues associated with the use of human fetal tissues and limited knowledge, the focus of such studies has shifted to other stem cells which could be differentiated into insulin-producing β cells [8-10]. Despite the advances in differentiation protocols of human stem cell populations toward pancreatic β cells, significant work remains to be done before cell transplantation of stem cell-derived β cells. Functional and structural differences between human fetal and adult pancreatic tissues have been known to some extent. Most of the reported studies are on fetal pancreas from the first trimester abortions where they have assessed for insulin release and transplantation studies [11]. The development of culture models of islets across the gestational ages (GA) would add benefit to enhance the understanding of the growth dynamics of the endocrine pancreas as these information details are limited [12-15]. A better knowledge of the developmental process would be helpful to deal with disease entities in a better way. The rationale behind performing this study was to see if viable islets could be isolated to study the islet characteristics at this GA.

## Materials and methods

Pancreatic specimen collection and preservation

The study was conducted after obtaining approval from the Institutional Review Board (IRB-12199, Ethics and Research Committee), of the Christian Medical College, Vellore, Tamil Nādu, India, 632002. This study was performed in line with the principles of the Declaration of Helsinki. The signed written informed consent was obtained from the participants by the investigators. The consent to publish the data was also obtained. The human fetal pancreas was obtained from the aborted fetuses within one hour of expulsion. The GA varied from 22 to 29 weeks. The fetuses with a maternal history of diabetes or gestational diabetes were excluded. Fetuses were collected and transported in a sterile container immediately after expulsion.

The dissection was carried out under appropriate sterile conditions to expose the pancreas and any pancreas with gross abnormality and autolytic changes were excluded. To improve the yield and quality of the tissue, the harvested pancreas was preserved in the cold preservation buffer, Hank’s balanced salt solution (HBSS, H4891 Sigma) supplemented with antibiotics and antifungal (penicillin-streptomycin -amphotericin, Sigma Aldrich Cat no: A5955). Each tissue was subdivided into two tissue blocks, one of which was used for morphologic studies and the other block was used to isolate the islets by enzyme digestion for functional assay.

Hematoxylin and eosin staining (H&E)

The pancreatic tissue bit thus obtained was dehydrated in alcohols of increasing concentration and embedded in paraffin. Serial sections of 4µM thickness were made with Leica Microtome (LEICA RM 2145, Germany) followed by H&E staining with a standard protocol of every 10th section. The sections were sequentially exposed to deparaffination, hydration with descending grades of alcohol (absolute, 90%, 70% Hayman, Witham F204325), and nuclear staining with hematoxylin (Qualigens Q39411) following counterstaining with eosin (Fisher Scientific, 39312).

DAB staining (3,3′-Diaminobenzidine)

4 µM sections were deparaffinized in the oven at 65˚C overnight, and the tissue sections were processed following the standard protocol for DAB staining. The slides were treated overnight with primary mouse anti-insulin antibody (Abcam K36aC10 ab6995, 1:500), primary rabbit polyclonal anti-glucagon antibody (Thermo Fisher Scientific, PA5 88091,1:200), and primary rabbit polyclonal anti-somatostatin antibody (Thermo Fisher Scientific, PA5 97200, 1:200) followed by treatment with the secondary antibodies, horseradish peroxidase-labeled goat anti-mouse (Thermo Fisher Scientific, Pierce 31430; 1:250) and goat anti-rabbit IgG peroxidase antibody (Sigma Aldrich A0545; 1:250) for an hour. The slides were stained with DAB chromogen (Thermo Fisher Scientific, Pierce Cat No: 34002) followed by counterstaining with Harris hematoxylin for one minute and were dehydrated with alcohol, cleared with Xylene (Qualigens, Thermo Fisher Scientific India, Q32297), and mounted with DPX ( Qualigens, Thermo Fisher Scientific India, Q18404) mounting.

Sequential double immunofluorescence staining of islets

A sequential double-staining immunofluorescence method was used to detect the expression of islet hormones, insulin, glucagon, somatostatin, and pancreatic polypeptide by confocal imaging. The tissue was fixed in 10% formalin (Sigma, F8775) and embedded in paraffin (sample: n=5). A 4µM section of the paraffin-embedded tissue was taken and was deparaffinized, rehydrated, and subjected to antigen retrieval. For deparaffination, the tissue sections were kept in the oven at 65˚C overnight, treated with xylene, and hydrated with descending grades of alcohol concentrations. Slides were transferred to 0.05M Tris-HCl (Invitrogen, AM9855G), and it was kept in a floating water bath at 92˚C for 20 minutes followed by treatment with cold distilled water. The tissue was further washed with PBS (Dulbecco's phosphate buffered saline, Invitrogen, AM9625) three times and 0.1% PBST (0.1% tritonX100 in PBS, Qualigens, India Cat no:10655)) three times. The tissues were treated with a protein block solution containing 1% BSA (bovine serum albumin-Hi Media MB083) and 6% fetal bovine serum (FBS, Cat No.10270106, GIBCO, Thermo Fischer Scientific, Waltham, MA) in PBS solution for 30 minutes. The slides were treated overnight with primary antibodies namely anti-glucagon, anti-somatostatin, and anti-pancreatic polypeptide antibody [ZRB939] (1:400 diluted in 2% BSA in PBS) followed by incubation with secondary antibody IgG (H+L) highly cross-adsorbed goat anti-rabbit IgG, Alexa Fluor® 488 (A11034,1:100 dilution in 2% BSA in PBS) for an hour. The slides were washed with PBST three times and treated with primary anti-insulin antibody for four hours at room temperature (25˚C) followed by incubation with secondary antibody IgG (H+L) highly cross-adsorbed goat anti-mouse, Alexa Fluor® 594, Invitrogen™ (1:100 dilution in PBS) for an hour. The slides were stained with nuclear stain DAPI (Thermo Fisher Scientific, Cat No:62248) for five minutes, and following a wash, the slides were mounted with 90% glycerol (Sigma Aldrich, 49781) in PBS. The images were acquired with an fv1000 model Olympus laser scanning confocal microscopy.

Morphological analysis and quantifications

Microscopic sections of islet cells were analyzed using a confocal microscope (fv1000 model Olympus laser scanning confocal microscopy) equipped with ultraviolet illumination and filters for blue, red, and green fluorescence. Images were captured with a color CCD camera (Zeiss) and recorded on the computer through software. For quantification, the nuclei were labeled with DAPI. Data were expressed as the percentage of hormone-producing cells (β and non-β cells) among total pancreatic cells as defined by DAPI nuclear staining or as the percentage of cells producing insulin, glucagon, somatostatin, and pancreatic polypeptide relative to the total hormone-producing cells in each pancreas section.

Fetal islet isolation and culture

The fetal pancreas was washed multiple times with the cold transport medium, HBSS. It was transferred to a small glass bowl with cold media and was cut into small pieces with sterile surgical scissors/scalpel blades. Multiple washes were given in between, and the wash buffer was pipetted out without losing the minced tissue. The fresh enzyme solution was prepared by dissolving 5 mg collagenase type XI (Sigma-Aldrich, C7657) in 2 ml of HBSS to achieve a concentration of 2.5 mg/ml, filtered with a 0.22-micron syringe filter (Minisart 16532-6UK), and then stored at 4°C. The prepared organ sample was transferred with a spatula or forceps to a 50 ml centrifuge tube with the freshly prepared enzyme solution and oxygenated before placing it in a shaking water bath (REMI RS B12, India). The water bath temperature was set at 37°C and was agitated gently at a rate of 60 rpm for 5-10 minutes to aid the digestion process. On completion of digestion (tissue volume less than 10%), collagenase activity was terminated by the addition of 15-20 ml of cold HBSS with 10% fetal bovine serum. The solution was placed on ice and allowed the cells to settle for 10 minutes, then aspirate off half the volume of the supernatant. Wash and centrifuge steps were repeated twice with 5 ml of wash buffer (HBSS +10% FBS followed by RPMI-1640+10% FBS). The samples were centrifuged for three minutes at 300-x g (REMI C-23, India). After a wash, cells were re-suspended in culture media containing RPMI-1640 (Sigma Aldrich, R8755) supplemented with 10% FBS and 4 mM glutamine (Sigma Aldrich, G7513), and the media was replaced every two days.

Insulin release assay

After overnight culture, the cell suspension was washed with phosphate-buffered saline, and 100 µl of cell suspension was incubated in 1 ml of Krebs-Ringer buffer containing either 2.8 mM glucose (low glucose stimulation) or 28 mM glucose (high glucose stimulation) at 37°C for 60 minutes. Cells were stimulated with low glucose followed by high glucose concentration. The conditioned medium was collected after each stimulation, centrifuged, and stored in -20 freezers. Insulin content in the supernatant was assayed by chemiluminescence immunoassay method (IMMULITE 2000 XPi, Siemens, manufacturers assay range 2.0-300.0 µIU/ml, conversion factor; µIU/ml x 1=mIU/l, µIU/ml X 7.217= pmol/L).

Statistical analysis

Data are presented as mean ±SD. Statistical analysis for differences between groups was analyzed using the parametric t-test. Differences were considered statistically significant in the case of p<0.05.

## Results

Pancreatic tissue specimens from human fetuses (n=5) were analyzed. The details of the fetuses are given in Table 1.

**Table 1:** Details of the human fetuses used for pancreatic islet isolations HF: human fetus, II: second trimester, III: third trimester, IUD: intrauterine death, NTD: neural tube defect

Sl no	GA (wks)	Associated anomaly
HF1	29/III	IUD
HF2	24/II	Renal agenesis
HF3	22/II	NTD
HF4	22+4/II	Anencephaly
HF5	24+5d/II	IUD

**Figure 1:**
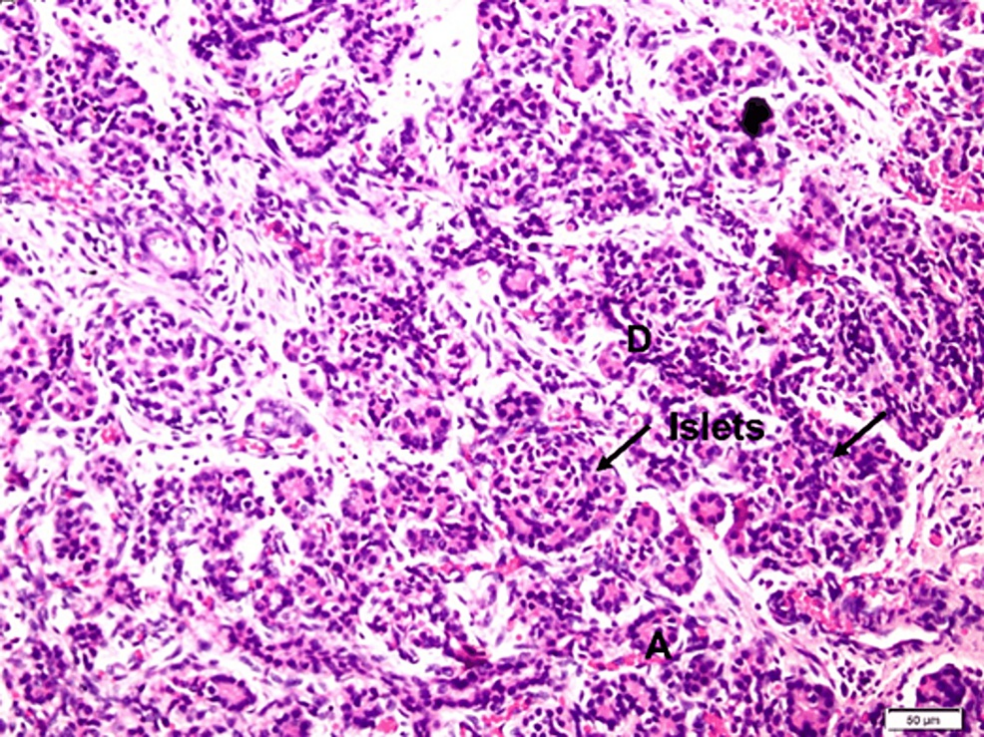
Histology of human fetal pancreas D: ducts, A: acini

For H&E, sections revealed some well-defined cell clusters like islets surrounded by developing acini and ill-defined mesenchymal connective tissue with capillaries (Figure 1).

**Figure 2:**
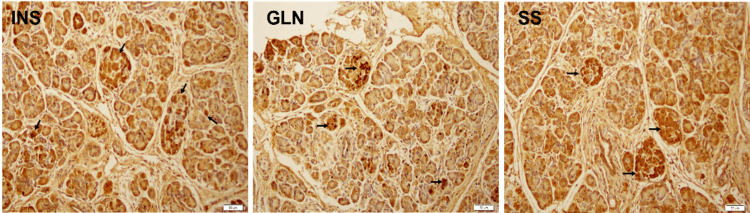
Chromogen staining of fetal pancreas sections demonstrates the presence of hormone-expressing cells in the islets-like clusters as well as single cells (seen as dark brown color). Lobulations were observed in the tissue sections INS: insulin, GLN: glucagon, SS: somatostatin

For the distribution of glucagon producing α cells, insulin-producing β cells, somatostatin-producing δ cells, pancreatic polypeptide-producing ϒcells in the developing islets of the human fetal pancreas, DAB and immunofluorescence staining confirmed islets and islets-like clusters as observed in H&E sections. Islets of varying sizes were observed, and some of the islets were identified as well-defined circumscribed spherical structures. In addition, randomly distributed hormone-positive single cells were also seen in the sections (Figure 2).

**Figure 3:**
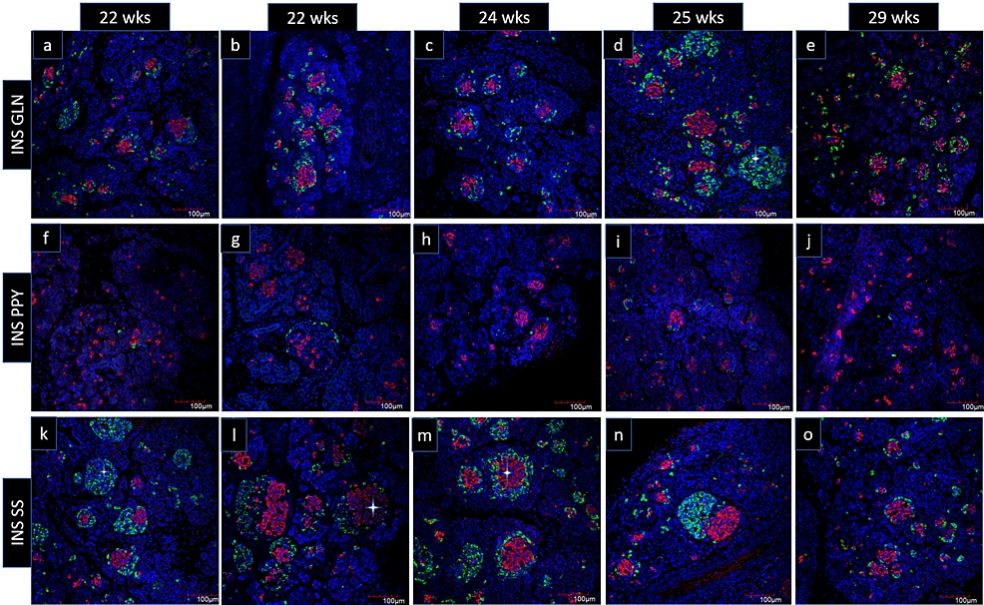
Confocal images of fetal pancreatic sections stained with islet markers (n=5). Demonstrates well-formed clusters, developing clusters, hormone +ve single cells, and homogenous clusters of single hormone-expressing cells. Images show developmental stages in islet formation INS: insulin (red), GLN: glucagon (green), SS: somatostatin (green), PPY: pancreatic polypeptide (green), DAPI: blue)

For the proportion of islet cells in the fetal pancreas with Immunofluorescence, the double immunostaining for insulin and glucagon showed insulin-secreting β cell expression in all sections of GA taken. These β cells mostly occupied the core of the islets. The α cells were similarly seen as abundantly expressed as β cells, and these cells were distributed at the periphery or mantle position of the islets around a central core of β cells (Figure 3a-3e). The double immunostaining for insulin and somatostatin showed the presence of δ cells in sufficient proportion like α cells. δ cells in clusters showed the same topographic arrangement as α cells and occupied the mantle position in a formed islet surrounding the central core of β cells (Figure 3k-3o). ϒ cells were least expressed and they occupied the periphery (Figure 3f-3j). However, we did not come across any co-expression of hormone-secreting cells during these GA. Homogenous clusters of α cells (Figure 3a) and δ cells (Figure 3k) were also evident in the stained sections.

**Figure 4:**
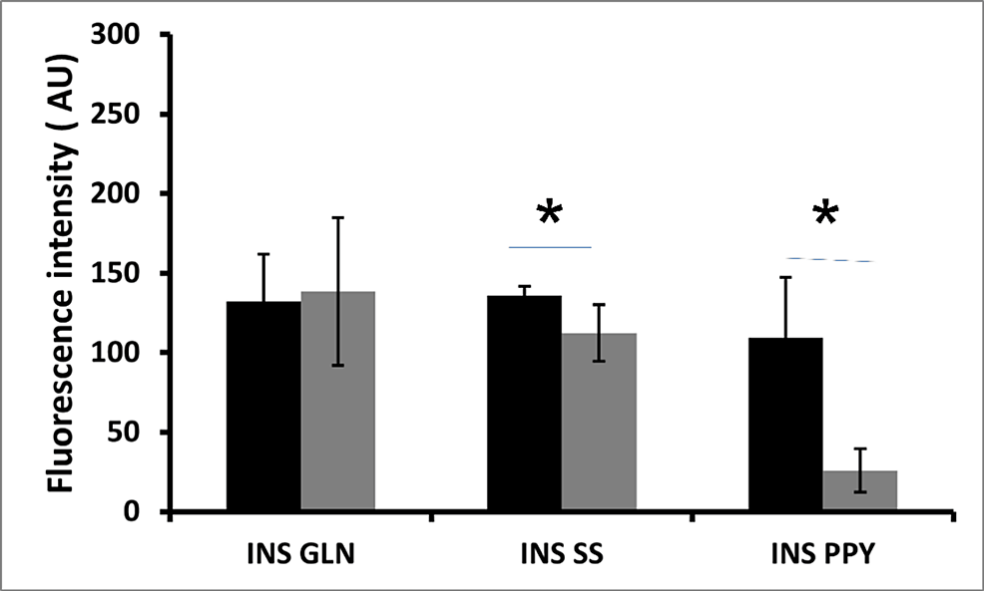
Mean ± SD values of fluorescence intensity exhibited by the endocrine cells of the pancreas when stained in a combination of β (insulin) with one of the non-β cell markers (glucagon, somatostatin, pancreatic polypeptide Y) AU: arbitrary unit, INS: insulin, GLN: glucagon, SS: somatostatin, PPY: pancreatic polypeptide Y INS/GLN 132.15 ±29.92, 138.45 ± 46.31; INS/SS 136.08 ± 5.84, 112.4 ±17.82; INS/PPY 109.43 ± 38.08, 25.86 ±13.54

For the quantification of α, β, δ, and ϒ from the immunohistochemistry section through confocal microscopy, to quantify the number of endocrine cells, pancreas sections from five fetuses were co-immunostained with antibodies directed against insulin, glucagon, somatostatin, and pancreatic polypeptide. Four separate fields for each pancreatic section (n=5) were used for computing the mean expression of each islet cell type (α, δ, ϒ) to β cells (Figure 4) in each section. The expression of β and α cells were found to be almost equivalent in distribution across GA included in the study. Y cells expression was the least.

**Figure 5:**
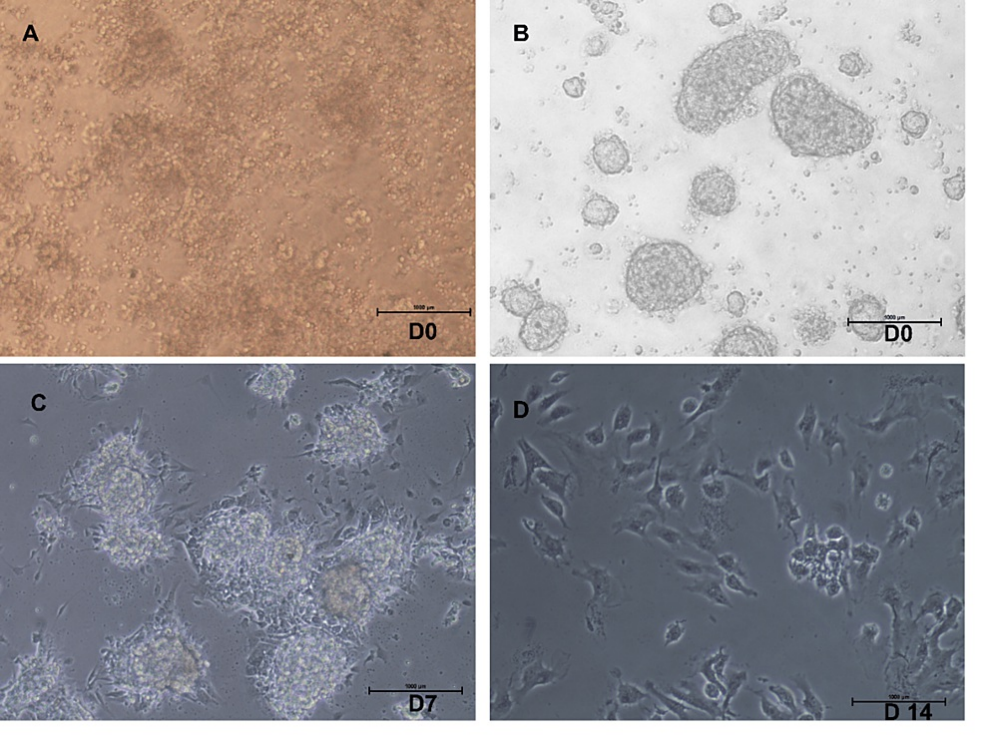
Isolated fetal islets in culture day 0, day 7, and day 14 (magnification x10)

For the formation of islet-like cell clusters in vitro, isolated islets were grown in culture for a week, and islet numbers were seen to reduce in the RPMI-FBS medium without supplements. Immediately after digestion, they were seen as single cells (Figure 5A), and formations/clusters start appearing after overnight culture (Figure 5B). Islet-like cell clusters observed after the digestion procedure were of different sizes. In some instances, where clusters were not evident on day 0, clusters were observed on the subsequent days. Some of the clusters were seen to be gradually increasing in size over the days (Figure 5B-5C). An outgrowth of elongated, spindle-shaped cells was observed on the cluster edges and shows adherence (Figure 5C). Islets form one of the fetuses when cultured for a longer time, and the major population of cells became elongated and showed fibroblast-like morphology over days in culture (Figure 5D).

**Figure 6:**
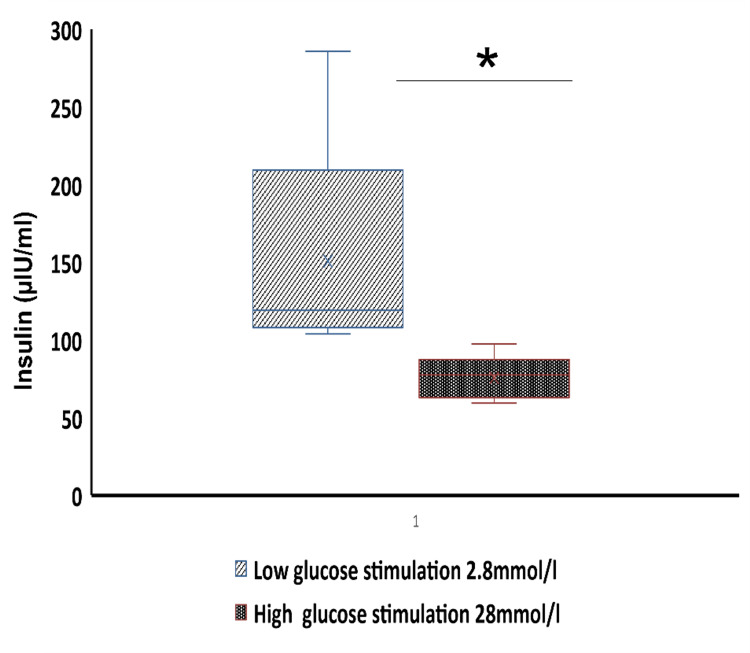
Glucose-stimulated insulin secretion of the isolated islets in culture (D1 islets). Islets were stimulated with 2.8 mM glucose or 28 mM glucose. The insulin levels in the conditioned medium were compared using a t-test. The insulin levels were expressed as μIU/ml * p<0.05

Isolated fetal islets were functional and released insulin. To evaluate the physiological maturation status of islet-like cell clusters insulin-releasing function was examined in response to a low and high concentration of glucose. The result showed that the response to first-time stimulation with low glucose (2.8 mmol/l) was strong, 150±76 µIU/ml, and when the same islets were immediately stimulated with high glucose concentration (28 mmol/l) resulted in reduced insulin secretion, 75±14 µIU/ml (Figure 6).

## Discussion

The human fetal islet model would be able to provide rich information on the developmental biology of the endocrine pancreas. The research on fetal islets holds promise because it could shed light on the unknown underlying mechanism of various pancreatic diseases. Though its suitability for transplantation is also a much sought-after application, ethical issues limit this line of investigation. The sufficient optimal functioning islets required remain essential in this regard. In the current study, we sought to investigate the functionality of pancreatic islets isolated from human fetuses of mid-GA. We also examined the developmental events in this particular GA by immunolabeling and confocal microscopy.

The cellular configuration of the adult human pancreas consists of 60% β cells and 30% α cells [16-17]. The β cells in adult humans remain interspersed with the α cells, specifically in the larger islets. Similarly, the mantle core pattern where β cells occupy the core with other cells in the periphery was observed in fetal islets also. Jeon et al. showed a cascade of endocrine cell clustering events in human fetal islets of 7-21 weeks of age with immunohistochemical analysis [12]. They observed the initial scattering of β and α cells, which gradually rearranged in a core-mantle relationship with glucagon cells occupying the periphery and insulin at the center by the 14th week of gestation. Our study also found a similar arrangement of δ and β cells being retained in fetuses of 22-29 weeks of GA. In addition, as shown in Figures 2-3, there were several hormones +ve single cells and homogenous cell clusters. It seems cell signaling cues drive the clustering of similar hormone-expressing cells before the formation of mature islets (Figure 3a-3i). The other possibility could be the proliferation of a single cell type to form homogenous cell clusters which has to be confirmed with proliferation markers. Once clusters of the homogenous cell population (β, α, δ) are formed, α and δ cell clusters move toward the β cell clusters, encircle, and incorporate the β cell mass eventually forming islets (Figure 3a, 3f, 3i). Thus, in developing islets, the core remains enriched with β cells while the α and δ cells move to the peripheral part. These findings highlight the significance of topographical arrangement in the form of islets rather than being separate cells or homogenous clusters. This affirms each cell in the islet has discrete regulatory functions through a complex intercellular network controlling paracrine signaling and neuronal control. In this regard, synthesizing β cells alone from the stem cells for the purpose of transplantation may have its own limitations.

Our result further demonstrated the relative composition of different endocrine cells in the pancreas across the developing fetuses by expressing their mean fluorescence intensity value. Unlike the adult pancreas, the α cells were the maximum among all the known endocrine cells, followed by β cells, in the fetal pancreas. The δ cells were also found to be equivalent to α cells and β cells. Reidel et al. showed the cellular composition of α cells and β cells in 9-21 weeks of GA [15]. They reported the presence of cells co-producing insulin and glucagon at nine weeks of fetal age. With increasing GA, there is a shift toward more α cells as compared to β cells, which seems to last till the 16th week of GA. The study observed an abundant expression of α cells even after 16 weeks as reported. An Indian study on the autopsy specimens of the adult non-diabetic pancreas documented a larger proportion of small islets with more β cells when compared to the American population, and this is hypothesized as one of the reasons for the increased susceptibility of Indians to diabetes mellitus [18].

Comparatively, only a few reports are available on the expression of somatostatin and pancreatic polypeptide cells in the human fetal pancreas. A higher density of δ and pancreatic polypeptide cells were reported in neonates and in the mid-term fetal pancreas than in the adult pancreas. As per Riedel, the δ and pancreatic polypeptide cells express independently at the age of 15th weeks, and the current study corroborates the same [15]. Interestingly, δ cells were found in abundance, and very few pancreatic polypeptide cells were seen in this gestational period, and this is in agreement with Clark et al., who likewise observed a relative increase in δ cells with the growing age of the fetus [19]. Somatostatin acts as a powerful inhibitor of the release of endocrine secretions, and, thus, high concentrations of δ cells might have some paracrine influence through inhibitory action required during development, which needs to be explored further. A recent study showed that in diabetic mice, neurogenin3+ ductal cells with somatostatin positive were found in increased numbers which further gives rise to β cells [14].

Exploring the expression profile markers in the fetal pancreas from the beginning to term with immunofluorescence would help us to understand the mechanism behind β cell genesis which could be targeted to trigger β cell neogenesis/proliferation in the adult pancreas. In vitro culture of these developing islets could witness interesting features captured on different days in culture. Cells after isolation aggregate in culture medium and develop into islets-like clusters of varying sizes. These evolving aggregates could be due to the cell cues present for aggregation and forming mature islets. At this juncture, the possibility of the presence of the progenitors in the cell pool could not be denied for the expanding or evolving islet-like clusters observed in vitro. The observed islet-like clusters were gradually increasing in size over days even in a medium devoid of specific supplements for growth. The addition of specific growth supplements could help to expand the precursor stem cell pool as studies have reported the expansion of human fetal progenitor cells found in 10-12 weeks of a fetus in a medium containing basic fibroblast growth factor and leukemia inhibitory factor which then differentiated to islet endocrine cells that aggregated to islet clusters [4]. The insulin release pattern of fetal islets was different from the adult islet response to low and high glucose stimulation. In adults, normal insulin release is more with high glucose stimulation than with low glucose stimulation. On the contrary, when the same set of fetal islets was exposed to low glucose followed by high glucose, insulin release was reduced. For first-time stimulation with low glucose, the associated observed insulin release could be due to release from the insulin stores. Immediate stimulation with high glucose reduced insulin release which could be due to exhausted stores and immature cell machinery to immediately synthesize new insulin to meet the demand.

The work reported in this study was performed only on the pancreases obtained from the second-trimester fetuses though the initial plan was to collect fetuses across all the trimesters which would have provided a more comprehensive understanding of the development of the endocrine pancreas. However, our study adds to existing evidence to understand their additional implications for islet neogenesis. Further in-depth proteomic and surfaceome analysis would help understand the beneficial effect of varying culture conditions on improving the viability of the fetal islets. Since the samples were available after varying ischemic times, compromised vascularity could have influenced their biological properties. Further evaluation using islets obtained from first- and third-trimester fetuses using improved media formulations would enhance further understanding of their potential therapeutic application in the field of islet transplantation.

## Conclusions

The study confirms the feasibility of isolating viable fetal islets, which could be grown in vitro, and reports the developmental stages observed in GA 22-29 weeks. We propound that the in vitro fetal pancreatic islets open tremendous opportunities to understand the normal developmental processes to extenuate pancreatic diseases and to discover new effective therapeutics focussing on in vivo β cell neogenesis.
